# Resistance to cisplatin does not affect sensitivity of human ovarian cancer cell lines to mifepristone cytotoxicity

**DOI:** 10.1186/1475-2867-9-4

**Published:** 2009-02-17

**Authors:** Elizabeth M Freeburg, Alicia A Goyeneche, Erin E Seidel, Carlos M Telleria

**Affiliations:** 1 Division of Basic Biomedical Sciences, Sanford School of Medicine of The University of South Dakota, Vermillion, SD 57069, USA

## Abstract

**Background:**

The prototypical antiprogestin mifepristone exhibits potent growth inhibition activity towards ovarian cancer cells *in vitro *and *in vivo*. The aim of this research was to establish whether mifepristone is capable of inhibiting cell proliferation and inducing apoptotic cell death regardless of the degree of sensitivity ovarian cancer cells exhibit to cisplatin.

**Methods:**

OV2008, OV2008/C13, A2780, A2780/CP70, Caov-3, and SK-OV-3 cell lines exhibiting a range of sensitivities to cisplatin were used. Growth inhibition, cell viability, and sub-diploid DNA content in response to treatment with escalating doses of either mifepristone or cisplatin were assessed by microcapillary cytometry. Apoptotic cell death was evaluated by measuring genomic DNA fragmentation and cleavage of caspase-3 and poly (ADP ribose) polymerase (PARP).

**Results:**

The sensitivities to cisplatin manifested by the cell lines were OV2008 > A2780 > Caov-3 > SK-OV-3 > OV2008/C13 > A2780/CP70. Mifepristone inhibited the growth of all six cell lines in a dose-related manner with IC_50s _ranging from ~6–12 μM and without significant correlation with the relative sensitivities the cells displayed for cisplatin. Moreover, at the highest concentration studied, mifepristone triggered apoptotic death in all six cell lines as evidenced by the increase in sub-diploid fragmented DNA content and cleavage of caspase-3 and of its downstream substrate PARP.

**Conclusion:**

Mifepristone is cytotoxic towards ovarian cancer cells independent of the sensitivity exhibited by the cells to cisplatin, displaying cytostatic effects at lower concentrations and lethal effects at higher concentrations. Mifepristone monotherapy emerges as a valuable therapeutic alternative for platinum-resistant ovarian cancers.

## Background

Current treatment for ovarian cancer begins with cytoreductive surgery followed by platinum-based chemotherapy [1-3]. However, long-term survival remains low because acquisition of resistance to platinum derivatives is a common feature for this disease, taking place with high frequency in patients with recurrent ovarian cancer [4-7]. This resistance is caused by the failure of a sufficient amount of platinum to reach the target DNA and/or the failure to achieve cell death after platinum-DNA adduct formation by development of more efficient DNA repair mechanisms or by increased tolerance to platinum-induced DNA damage [8,9]. Hence, finding new treatment alternatives for platinum-insensitive ovarian cancers is of critical importance.

In preclinical studies previously conducted in our laboratory, the antiprogestin steroid mifepristone was found to be highly effective as a single agent *in vitro *and *in vivo *abrogating growth of human epithelial ovarian cancer cells [10]. We demonstrated that the growth inhibitory effect of mifepristone on ovarian cancer cells was associated with inhibition of DNA synthesis, down-regulation of transcription factor E2F1 needed for S phase progression, and inhibition of the activity of cell cycle regulatory kinase, cyclin dependent kinase 2 (Cdk2). This is likely due to increased association of Cdk2 with the Cdk inhibitors p21^cip1 ^and p27^kip1 ^which are greatly up-regulated in response to the drug. All these molecular events downstream of mifepristone action lead to blockage of the cell cycle at the G1-to-S phase transition [10]. In the same study it was observed that mifepristone displayed similar growth inhibition potency among SK-OV-3, OV2008, and Caov-3 cell lines [10]. To note is that whereas OV2008 cells were reported as being highly sensitive to cisplatin [11], SK-OV-3 cells were originally obtained from a patient with intrinsic resistance to clinically achievable doses of cisplatin [12], and Caov-3 cells were reported to be resistant to cisplatin [13,14]. Based on this information, it is reasonable to speculate with the possibility that mifepristone may be useful in abrogating ovarian cancer cell growth irrespective of the sensitivity the cells display for cisplatin.

The tumor suppressor p53 encodes for a transcription factor which is involved in a multiplicity of cellular functions including cell cycle [15,16], cell death [17,18], cell differentiation [19], and DNA damage [18,20] and repair [21,22] pathways. In ovarian cancer, mutations in the p53 gene correlate with resistance to platinum-based chemotherapy and shortened survival [23]. In addition, p53 is non-functional in 70% of ovarian tumors [24], whereas preclinical studies suggest this tumor suppressor is a determinant of cisplatin sensitivity in ovarian cancer cells [25-27].

It is reasonable to contemplate the possibility that if both the sensitivity to platinum and the p53 genetic background of ovarian cancer cells do not condition their response to the growth inhibition activity of mifepristone, such findings would have great clinical relevance. Thus, in the present work we first set out to study the growth inhibition activity of mifepristone among ovarian cancer cell lines having different sensitivities to platinum derivatives. We studied the action of mifepristone not only in ovarian cancer cells with different genetic backgrounds, but also among ovarian cancer cell line pairs consisting of cisplatin-sensitive parental lines and stable cisplatin-resistant sublines derived by *in vitro *selection with stepwise exposure to increasing doses of cisplatin. In addition, because of the differences in the p53 genetic status of the cell lines studied, the experiments indirectly allowed us to provide evidence as to whether the p53 genetic background impacts the response of the ovarian cancer cells to mifepristone.

## Methods

### Cell lines and drugs

The human ovarian carcinoma cell lines, OV2008, OV2008/C13, A2780, and A2780/CP70, were obtained from Dr. Stephen Howell (University of California, San Diego) and were maintained in RPMI 1640 (Mediatech, Herndon, VA) supplemented with 5% heat inactivated FBS (Atlanta Biologicals, Lawrencenville, GA) and 10 mM HEPES (Mediatech), 4 mM L-glutamine (Mediatech), 1 mM sodium pyruvate (Mediatech), 1 X non-essential amino acids (Mediatech), 100 IU penicillin (Mediatech) and 100 μg/ml streptomycin (Mediatech). Caov-3 and SK-OV-3 ovarian cancer cells were obtained from the American Type Culture Collection (ATCC, Manassas, VA) and were routinely maintained in RPMI 1640 (Mediatech) supplemented with 5% FBS (Atlanta Biologicals), 10 mM HEPES (Mediatech), 4 mM L-glutamine (Mediatech), 0.45% D (+) glucose (Sigma Chemical Company, St. Louis, MO), 1 mM sodium pyruvate (Mediatech), 1 X non-essential amino acids (Mediatech), 100 IU penicillin (Mediatech), 100 μg/ml streptomycin (Mediatech), and 0.01 mg/ml human insulin (Roche, Indianapolis, IN). All cell lines were cultured at 37°C in a humidified atmosphere in the presence of 5% CO_2_.

The stock of mifepristone (Sigma) was 116.5 mM solution in DMSO. The maximal concentration of DMSO was 0.02% (v/v). The stock of cisplatin (cis-diamminedichloroplatinum II) (Sigma) was 3 mM solution in 0.9% NaCl. Cells were exposed to cisplatin for only 1 h. Thereafter, the medium was replaced with fresh cisplatin-free medium. Cells exposed to mifepristone were cultured in the continuous presence of the drug throughout the studies.

### Cell proliferation and viability

Triplicate cultures were trypsinized, pelleted by centrifugation at 500 *g *for 5 min, and washed with PBS. The cells were resuspended in ViaCount reagent (Guava Technologies, Hayward, CA) and studied using the Guava ViaCount application in the Guava EasyCyte Mini microcapillary cytometer (Guava Technologies). This assay provides an absolute cell count and viability data on a cell suspension, automating results like cell counts in a hemocytometer chamber with the trypan blue dye exclusion method for assessing cell viability. The cells are drawn into a capillary flow cell of known dimensions at a precisely controlled rate for measured periods of time. Absolute cell counts are obtained by knowing the exact sampling volumes. Viable and non-viable cells are assessed by the differential permeability of two DNA-binding dyes in the reagent. One dye is membrane permeable and stains all nucleated cells. The other dye only penetrates cells with compromised membrane integrity (i.e. non-viable cells). The data are acquired and analyzed using the CytoSoft 4.1 software (Guava Technologies).

For the cells treated with either cisplatin or mifepristone, three inhibition concentration 50% or IC_50 _values averaged for each cell line and drug were obtained. The IC_50 _values were calculated using the drug interaction software (Calcusyn, Biosoft, Cambridge, UK), which was designed to study drug interaction and calculates the median effective dose, Dm, which is analogous to the IC_50_.

### Determination of sub-G1 DNA content

After treatment, cells were trypsinized, pelleted by centrifugation at 500 *g *for 5 min, washed with PBS, and fixed with 4% paraformaldehyde. Cells were once again washed with PBS and pelleted by centrifugation at 500 *g *for 5 min. Then, approximately 100,000–200,000 cells were resuspended in 200 μl of cell cycle buffer [3.8 mM sodium citrate (Sigma), 7 U/ml RNase A (Sigma), 0.1% (v/v) Triton X-100 (Sigma), and 0.05 mg/ml propidium iodide (Sigma)] at a concentration of 500–1000 cells/μl. Cells were analyzed for the capacity of their DNA to bind propidium iodide utilizing the Guava EasyCyte Mini microcapillary cytometer and the cell cycle application of the CytoSoft 4.1 software (Guava Technologies), with special emphasis on the analysis of the cellular fragments with hypodiploid DNA content.

### SDS-PAGE and Western blotting

Cells were scraped, pelleted, washed twice with PBS, and lysed by the addition of two volumes of radioimmunoprecipitation assay buffer (RIPA) containing 50 mM Tris-HCl (pH 7.4), 150 mM NaCl, 1% NP-40 (Sigma), 0.25% sodium deoxycholate (Sigma), 1 mM EDTA, 1 mM PMSF (Sigma), 1 μg/ml pepstatin (Sigma), 1 mM orthovanadate (Sigma) and 1 mM sodium fluoride (Sigma). Cells were disrupted by passing them through a 21 gauge needle, and gently rocked on ice for 30 min. Lysates were centrifuged at 16,000 *g *for 15 min at 4°C, and the supernatant was considered the whole cell extract, which was assayed for protein content by using the bicinchoninic acid method (BCA; Pierce, Rockford, IL). Equivalent amounts of protein (50 μg) per point were loaded in 12% (w/v) acrylamide gels, subjected to SDS-PAGE and transferred to PVDF membranes. The blots were blocked in 5% (v/v) nonfat milk in TBS containing 0.1% (v/v) Tween 20 (T). Blots were then probed overnight with primary antibodies against poly (ADP-ribose) polymerase (PARP) (#9542; 1:1000; Cell Signaling Technologies, Danvers, MA) or caspase-3 (#9662; 1:1000; Cell Signaling). The membranes were washed 3 × 5 min in TBS-T and incubated with 1: 10,000 dilution of peroxidase-conjugate secondary antibody (#111-035-003; Jackson ImmunoResearch Laboratories, West Grove, PA) for 30 min at room temperature. The blots were again washed, developed by chemiluminescence, and exposed to radiographic film. Blots were also probed with an antibody directed against β-Actin (clone AC-15; 1:20,000; Sigma) to control for protein loading.

### DNA fragmentation

Floating and adherent cells were pelleted and digested overnight at 50°C in a buffer composed of 100 mM NaCl, 10 mM Tris HCl (pH 8.0), 25 mM EDTA (pH 8.0), 0.5% SDS and 0.1 mg/ml proteinase K (Life Technologies, Rockville, MD). The genomic DNA was extracted from the digested cells with phenol/chloroform/isoamyl alcohol (25:24:1, v/v/v), precipitated, and digested for 60 min at 37°C with 1 μg/ml ribonuclease (deoxyribonuclease-free; Roche, Indianapolis, IN). After extraction and precipitation, an equal amount of DNA for each sample (2 μg) was separated by electrophoresis on a 2.5% agarose gel, impregnated with SYBR Gold nucleic acid gel stain (Molecular Probes, Eugene, OR) and photographed with the Amersham Typhoon Fluorescence imaging system (Amersham Biosciences Corp., Piscataway, NJ). A 100 bp DNA ladder (Promega, Madison, WI) was utilized for determining the size of the fragments of DNA.

### Statistical analysis

All data are reported as means ± SEM, and statistical significance was defined as *p *< 0.05. To compare cell growth, cell viability, sub-diploid DNA distribution, and IC_50 _values, one-way ANOVA followed by the Newman-Keuls' multiple comparison test or two-way ANOVA followed by the Bonferroni's multiple comparison test were used as appropriate. In addition, a Pearson correlation statistical test was utilized to quantify the degree to which the IC_50s _for mifepristone and cisplatin for each cell line relate to one another.

## Results

### Growth inhibition and lethality of cisplatin towards ovarian cancer cells of similar genetic backgrounds but different platinum sensitivities

**Figure 1:**
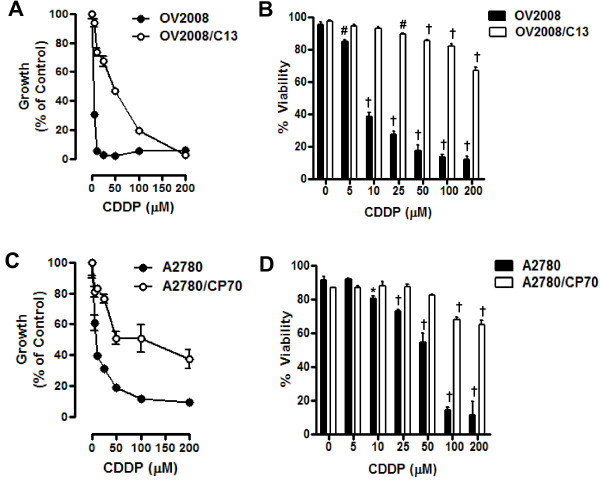
**Growth inhibition and lethality of cisplatin towards OV2008, OV2008/C13, A2780 and A2780/CP70 cells**. Cells were cultured in the presence of the indicated concentrations of cisplatin for 1 h and maintained in cisplatin-free medium for 3 days. (A) and (C), the total number of cells was recorded at the beginning of the experiment and after 3 days of treatment. The difference between number of cells in vehicle-treated controls at 0 h and after 3 days of culture was considered to be 100%. The growth of the treated groups is expressed as percentage of control. The experiment was repeated three times with different cell line passages to calculate the IC_50 _(see averaged data on Table 1). Depicted are representative experiments. (B) and (D), after treatment, viability was assessed by microcapillary cytometry. Results are the average of triplicate counts ± SEM. *p < 0.05, ^#^*p *< 0.01 and ^†^*p *< 0.001 vs. control (0 μM cisplatin) for the respective cell line.

**Figure 2:**
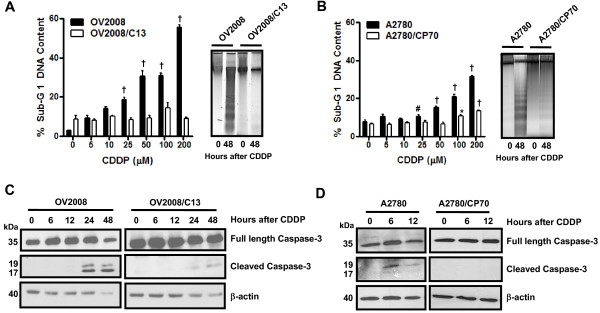
**Sub-G1 DNA Content, DNA fragmentation, and cleavage of caspase-3 in OV2008, OV2008/C13, A2780 and A2780/CP70 cells treated with cisplatin**. (A, left panel) OV2008 and OV2008/C13 cells were treated with or without the depicted concentrations of cisplatin for 1 h. Seventy-two hours later, floating and adherent cells were collected, fixed with 4% paraformaldehyde, stained with propidium iodide, and analyzed by microcytometric analysis using cell cycle software. (A, right panel) In a similar experiment, cells were exposed to a fixed 100 μM cisplatin concentration for 1 h and were harvested 48 h later; genomic DNA was isolated, separated by electrophoresis on a 2% agarose gel, impregnated with SYBR Gold nucleic acid stain, examined with an ultraviolet transilluminator, and photographed. (B) This graph depicts results from experiments similar to those shown in (A), but using A2780 and A2780/CP70 cells instead. *p < 0.05, ^#^*p *< 0.01 and ^†^*p *< 0.001 vs. control (0 μM cisplatin) for the respective cell line. (C and D) Cells were harvested at the indicated times after 1-h exposure to 100 μM cisplatin, whole cell proteins were isolated, equivalent amounts of proteins were electrophoresed, transferred to a PVDF membrane, and exposed to anti-caspase-3 or β-actin antibodies.

We utilized two pairs of human ovarian cancer cell lines, each pair consisting of a cisplatin-sensitive parental line and a stably cisplatin-resistant subline derived by *in vitro *selection with cisplatin. The resistance of these cell lines to cisplatin, determined using a clonogenic survival assay and a 1-h exposure to cisplatin, has been previously reported to be 8.1 fold for A2780/CP70 vs. A2780, and 5.7 fold for OV2008/C13 vs. OV2008 [11]. The different sensitivity of these ovarian cancer cell lines to cisplatin was further confirmed in our laboratory. Cells were exposed to 0, 5, 10, 25, 50, 100, or 200 μM cisplatin for 1 h, and the culture was continued for 72 h in cisplatin-free media. At the end of the experiment, non-adherent and adherent cells were harvested, and their number and viability was assessed by microcytometry. The experiment was repeated three times utilizing different cell line passages. The cisplatin IC_50s _calculated for the cell lines were, as expected, significantly lower for OV2008 cells when compared to OV2008/C13 cells, and significantly lower for A2780 cells when compared to A2780/CP70 cells (Figure 1A and 1C, and Table 1). These results confirm the predictions that OV2008/C13 and A2780/CP70 are less sensitive to cisplatin than their sisters OV2008 and A2780, respectively. The different sensitivity to cisplatin was also manifested in the significant reduction in viability of OV2008 and A2780 cells in response to cisplatin exposure when compared to the response of OV2008/C13 and A2780/CP70 cells, respectively (Figure 1B and 1D). Furthermore, sub-G1 DNA content, which is usually associated with apoptotic cell death [28], increased more significantly in response to cisplatin in OV2008 and A2780 cells when compared to the levels observed in OV2008/C13 and A2780/CP70 cells (Figure 2, left panels in A and B). The apoptotic nature of the cell death process triggered by cisplatin was confirmed by fragmentation of the genomic DNA which was substantially more evident in OV2008 and A2780 cells when compared to the DNA fragmentation observed in OV2008/C13 and A2780/CP70 cells in response to treatment with 100 μM cisplatin for 1 h (Figure 2, right panels in A and B). Finally, the different response to cisplatin was highlighted by the cleavage of the marker of apoptosis, 35 kDa procaspase-3, to presumably active 19 and 17 kDa fragments, which was evident in OV2008 and A2780 cells, but not in OV2008/C13 and A2780/CP70 cells (Figure 2C and 2D). Together, results in Figures 1 and 2 confirm that the two sister ovarian cancer cell line pairs, OV2008 and OV2008/C13, and A2780 and A2780/CP70, although carrying similar genetic backgrounds, responded very differently to cisplatin-induced lethality.

**Table 1:** IC_50s _for mifepristone and IC_50s _for cisplatin in various ovarian cancer cell lines of different p53 genetic backgrounds and platinum sensitivities.

**Cell line**	**IC_50 _MF (μM)**	**IC_50_CDDP (μM)**	**Reported p53 status**
**OV2008**	8.8 ± 0.8 (5)	0.2 ± 0.1 (3)	wt [30]
**OV2008/C13**	8.1 ± 0.6 (3)	49.8 ± 10 (3)	wt [30,38]
**A2780**	5.9 ± 0.6 (3)	6.0 ± 1.3 (3)	wt [39,40,52]
**A2780/CP70**	11.7 ± 0.6 (3)*	55.0 ± 11 (3)	mut [30,38-40]
**SK-OV-3**	12.6 ± 0.1 (3)*	18.1 ± 0.6 (3)	mut [31,33,34]
**Caov-3**	7.4 ± 1.8 (3)	15.2 ± 1.4 (3)	mut [31,32]

**Figure 3:**
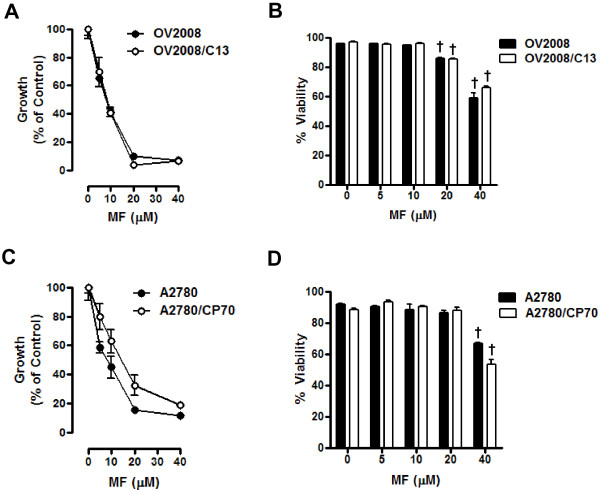
**Growth inhibition and lethality of mifepristone towards OV2008, OV2008/C13, A2780 and A2780/CP70 cells**. Cells were cultured in the presence of the indicated concentrations of mifepristone for 72 h. (A) and (C), the total number of cells was recorded at the beginning of the experiment and after the 3 days of treatment. The difference between number of cells in vehicle-treated controls at 0 h and after 3 days of culture was considered to be 100%. The growth of the treated groups is expressed as percentage of control. The experiment was repeated three times with different cell line passages to calculate the IC_50 _(see Table 1 for averaged data). Depicted are representative experiments. (B) and (D), after treatment, viability was assessed by microcapillary cytometry. Results are the average of triplicate counts ± SEM. ^†^*p *< 0.001 vs. control (0 μM mifepristone) for the respective cell line.

Cells were treated with mifepristone (MF) or cisplatin (CDDP) as described in Figure 1A and 1C, Figure 3A and 3C, and Figure 5A, to calculate the concentration of the drugs needed to achieve 50% growth inhibition (IC_50_). The experiments were repeated at least three times with different cellular passages, each time in triplicates. The numbers in parentheses indicate the number of experiments carried out for each cell line. IC_50s _are expressed as the mean ± SEM. *p < 0.05 compared with the other cell lines treated with mifepristone. The numbers in brackets indicate references that reported the p53 genetic status of the depicted cell lines.

### Growth inhibition and lethality of mifepristone towards ovarian cancer cells occurs regardless of cisplatin sensitivities

**Figure 4:**
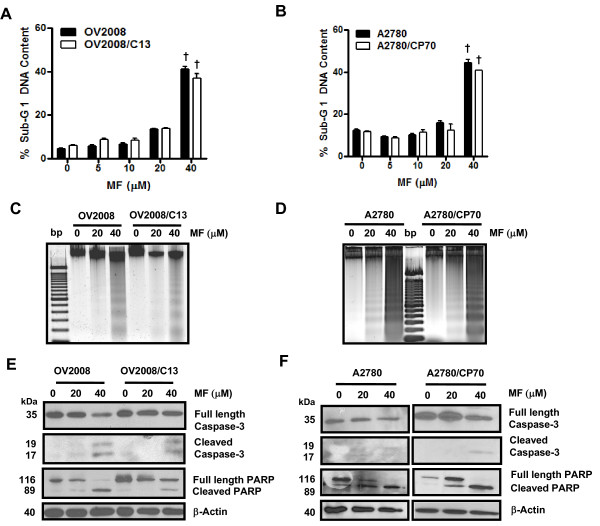
**Sub-G1 DNA Content, DNA fragmentation, and activation of caspase-3 in OV2008, OV2008/C13, A2780 and A2780/CP70 cells treated with mifepristone**. OV2008 and OV2008/C13 cells (A) or A2780 and A2780/CP70 cells (B) were treated with or without the depicted concentrations of mifepristone. Seventy-two hours later, floating and adherent cells were collected, fixed with 4% paraformaldehyde, stained with propidium iodide, and analyzed by microcytometric analysis using cell cycle software. ^†^*p *< 0.001 vs. control (0 μM mifepristone). In a similar experiment OV2008 and OV2008/C13 cells (C) or A2780 and A2780/CP70 cells (D) were exposed to the various concentrations of mifepristone. Cells were harvested 72 h later, and genomic DNA was isolated, separated by electrophoresis on a 2% agarose gel, impregnated with SYBR Gold nucleic acid stain, examined with an ultraviolet transilluminator, and photographed. In another experiment, OV2008 and OV2008/C13 cells (E) or A2780 and A2780/CP70 cells (F) were exposed to various concentrations of mifepristone and harvested 72 h later. Whole cell proteins were isolated, equivalent amounts of proteins were electrophoresed, transferred to a PVDF membrane, and exposed to anti-caspase-3, anti-PARP, or β-actin antibodies.

To study if the cell pairs OV2008 and OV2008/C13, and A2780 and A2780/CP70 responded similarly to the toxicity of mifepristone, the cells were plated and exposed for 72 h to 0, 5, 10, 20 or 40 μM mifepristone. The antiprogestin growth inhibited OV2008 and OV2008/C13 similarly, with IC_50s _that were indistinguishable from one another (Figure 3A and Table 1). In the case of the A2780 and A2780/CP70 pair, mifepristone blocked growth in a dose-dependent manner; however, the IC_50 _for A2780 was significantly lower than that for A2780/CP70 (Figure 3C and Table 1). Mifepristone was cytostatic to all cell lines studied at concentrations ranging from 0–10 μM, but shows some lethality towards the OV2008 and OV2008/C13 pair at the 20 μM concentration, which was more evident at the concentration of 40 μM (Figure 3B). In the A2780 and A2780/CP70 pair, the lethality of mifepristone was manifested only at the concentration of 40 μM (Figure 3D). The lethality induced by mifepristone to the ovarian cancer cell lines was further confirmed by the detection of cellular particles containing hypodiploid DNA content in coincidence with the treatment done with 40 μM of the drug in the four cell lines studied (Figure 4A and 4B), without apparent differences in the behavior of the cisplatin-sensitive versus the cisplatin-resistant sibling cells. The presence of cellular particles with deficient DNA content as represented by the sub-G1 region of the cell cycle histogram in propidium iodide-stained cells, suggested that cell death occurred by apoptosis [28]. This was confirmed by the detection of the characteristic ladder of DNA denoting DNA fragmentation in the four cell lines exposed to 40 μM mifepristone (Figure 4C and 4D). Finally, the lethality induced by mifepristone in all cell lines, without apparent distinction of platinum-sensitivities, was associated with another marker of apoptotic death, the cleavage of the caspase-3 target PARP from the full length 116 kDa form to a 89 kDa fragment [29] (Figure 4E and 4F). In OV2008 and OV2008/C13 cells, cleavage of caspase-3 was observed in parallel to the cleavage of its target PARP (Figure 4E). In A2780 and A2780/CP70 cells, the decline in full length caspase-3 followed the cleavage of PARP, whereas the cleaved caspase-3 fragments, although present, were less evident (Figure 4F). Altogether, results in Figures 3 and 4 demonstrate that despite the differential sensitivity to cisplatin displayed by the two pairs of sister ovarian cancer cell lines, they responded to the toxicity of mifepristone in a similar fashion. Mifepristone was cytostatic at lower concentrations and lethal at higher concentrations in both, cisplatin-sensitive OV2008 and A2780 cells, and cisplatin-resistant OV2008/C13 and A2780/CP70 cells.

### Mifepristone inhibits growth and kills ovarian cancer cells of different p53 genetic backgrounds in association with genomic DNA fragmentation and activation of caspase-3

**Figure 5:**
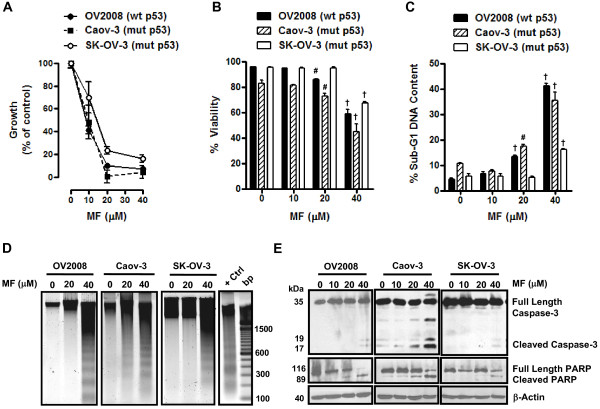
**Effect of mifepristone on growth, viability, fragmentation of genomic DNA, and on expression of caspase-3 and of its downstream substrate poly (ADP) ribose polymerase (PARP) in OV2008, Caov-3, and SK-OV-3 cells**. Cells were plated in equal number, allowed to attach to the plate surface for 24 h, and were then treated with either vehicle (DMSO) or the indicated doses of mifepristone in cell specific culture media for 72 h. Cells were then trypsinized, stained, and counted by microcapillary cytometry. The experiments were repeated at least three times in triplicates for each of the doses tested. A representative experiment is shown for each of the cell lines. (A) The total number of cells was recorded at the beginning of the experiment and after 3 days of treatment. The difference between number of cells in vehicle-treated controls at 0 h and after 3 days of culture was considered to be 100%. The growth of the treated groups is expressed as percentage of control. (B) This experiment was similar to that described in previous panel. Following the plating and treatment protocol, cells were collected after 72 h and viable cells were recorded by microcytometry using the Guava ViaCount application. Bars, mean ± SEM. ^#^*p *< 0.01 and ^†^*p *< 0.001 when compared to the control (0 μM mifepristone) for each cell line. (C) Following plating and treatment protocol, cells were collected after 72 h, fixed in 4% paraformaldehyde, stained with propidium iodide, and analyzed by cytometry using the Guava cell cycle application. Bars, mean ± SEM. ^#^*p *< 0.01; ^†^*p *< 0.001 when compared to 0 μM mifepristone. (D) Genomic DNA was isolated and separated by electrophoresis on a 2% agarose gel, impregnated with SYBR Gold nucleic acid stain, examined with an ultraviolet transilluminator, and photographed with the Amersham Typhoon fluorescence imaging system. A 100 base pair marker and a positive control (+ Ctrl) of fragmented DNA generated by treating OV2008 cells with cisplatin were run in parallel. (E) Cells were treated with the indicated concentrations of mifepristone for 72 h, whole cell proteins were isolated, electrophoresed, electrotransferred to a PVDF membrane, and exposed to anti-caspase-3 and anti-PARP antibodies. β-Actin was used as loading control.

To explore whether mifepristone can inhibit the growth of ovarian cancer cells regardless of their p53 genetic statuses, we studied the response to mifepristone of OV2008 cells that express wild type p53 [30], Caov-3 cells which express an mRNA carrying a point mutation that results in a chain termination signal likely to generate a truncated peptide not detected by Western blot [31,32], and SK-OV-3 cells carrying a deletion of a single nucleotide as a consequence of which no p53 mRNA transcripts are expressed [31,33,34]. The three cell lines were treated or not treated with increasing doses of mifepristone for 72 h. At the end of the experiment, cells were evaluated and analyzed by microcapillary cytometry for cell number, cell viability, sub-G1 DNA content, genomic DNA fragmentation and expression of caspase-3 and PARP. Results shown in Figure 5A illustrate that all cell lines were growth inhibited by mifepristone in a dose-dependent manner. At the highest dose utilized (40 μM) mifepristone inhibited the growth of the three cell lines greater than 90%. SK-OV-3 cells were found to have a mifepristone IC_50 _concentration significantly higher than that of OV2008 and Caov-3 cells, both of which had similar mifepristone IC_50s _(Figure 5A and Table 1). Mifepristone showed some lethality towards OV2008 and Caov-3 cells at the 20 μM concentration, but not on SK-OV-3 cells. At the concentration of 40 μM, mifepristone very strongly reduced the viability of the three cell lines (Figure 5B). This lethal effect of mifepristone was reflected by the increased percentage of cellular particles with hypodiploid DNA content (Figure 5C), and fragmentation of the genomic DNA in a characteristic ladder (Figure 5D). Finally, the cell death process triggered by mifepristone in the three ovarian cancer cell lines was associated with activation of caspase-3 marked by the cleavage of full-length pro-caspase-3 into 17 and 19 kDa fragments, which paralleled the cleavage of the caspase-3 downstream substrate PARP to the 89 kDa fragment (Figure 5E).

### The response to mifepristone-induced cytotoxicity does not correlate with the sensitivity the ovarian cancer cells displayed to cisplatin

Because we analyzed the response to six different ovarian cancer cell lines to cisplatin and mifepristone in parallel, with experiments done separately three times with three different cell line passages, we considered it valid to analyze whether or not the IC_50s _found for mifepristone and the IC_50s _found for cisplatin among the cell lines relate to one another. To answer this question, a Pearson's correlation study statistics was performed to establish the relationship between the IC_50 _values obtained for each drug. Analysis of data shown in Table 1 indicates the lack of statistically significant difference in the response of the cells to mifepristone despite the broad ranges of responses to cisplatin (r = 0.3931; p > 0.05). Whereas the IC_50s _for cisplatin ranged from < 1 μM to as high as 55 μM, the IC_50s _for mifepristone ranged only from ~6–12 μM.

## Discussion

Mifepristone inhibited ovarian cancer cell growth despite the fact that the cell lines studied had similar genetic backgrounds but very different sensitivities to cisplatin acquired by *in vitro *selection of clones upon challenges with cisplatin. The cisplatin-sensitive OV2008 cells were originally established from a patient with serous cystoadenocarcinoma of the ovary [35,36], and the cisplatin-resistant OV2008/C13 cells were generated from OV2008 cells by monthly *in vitro *selection with 1 μM cisplatin [35]. The cisplatin-sensitive A2780 cells were originally obtained from an untreated patient and were made resistant to cisplatin *in vitro *by stepwise exposure to a final concentration of 20 μM cisplatin and identified as A2870/CP70 [37]. Whereas the cisplatin-resistant OV2008/C13 cells maintained p53 wild type expression during the process of *in vitro *challenge with cisplatin when compared to their sister OV2008 cells [30,38], that was not the case for the A2780/CP70 cells that acquired a p53 mutation during such an *in vitro *selection process [39,40]. This phenomenon is not surprising as it has been shown that there is a survival advantage of p53 mutant cells in the presence of genotoxic cisplatin [27,41]. The tumor suppressor p53 appears to be a determinant of cisplatin sensitivity since mutant p53 status is often associated with cisplatin insensitivity [26], whereas reintroduction of wild type p53 via adenovirus gene transfer into A2780/CP70 cells significantly sensitized these cells to cisplatin lethality [42]. However, it has also been reported that the mutation of the p53 gene in the A2870/CP70 cells does not lead to expression of a mutant p53 protein when the cells are challenged with cisplatin; conversely, when the challenge is with ionizing radiation, they do express increasing levels of a mutant p53 protein capable of up-regulating the p53-target gene p21^cip1^, and of showing transcriptional activity in a functional assay [39]. Thus, it is the signal transduction pathway connecting cisplatin action and p53 gene expression what appears to be impaired in A2780/CP70 cells. This might be related to the fact that whereas OV2008 and OV2008/C13 cells have similar mifepristone IC_50s_, the mifepristone IC_50 _of A2780/CP70 was significantly higher than that of A2780 cells. Yet, although with slightly different potency, it can be concluded that mifepristone was very effective at blocking growth in the four ovarian cancer cell line pairs investigated considering the broad range of cisplatin IC_50s _(< 1 to 55 μM) and the narrow range of mifepristone IC_50s _(~6–12 μM).

Notably, mifepristone was cytostatic at concentrations lower than 20 μM, but it was lethal at concentrations higher than 20 μM. The cytostatic nature of concentrations of mifepristone up to 20 μM towards ovarian cancer cells was previously shown in our laboratory by demonstrating the reversibility of the growth inhibition effect when the drug was removed from the culture [10]. Furthermore, we have recently demonstrated that intertwining cytostatic concentrations of mifepristone in between courses of lethal cisplatin chemotherapy not only resulted in an efficacious strategy to prevent repopulation of cancer cells in between lethal platinum treatment intervals, but it also potentiated cisplatin killing efficacy [43]. Interestingly, however, in the present work we are showing that concentrations of mifepristone higher than that needed to achieve cytostasis are *per se *lethal to ovarian cancer cells. This lethality was illustrated by the reduced viability of the cells, the increase in cellular particles with hypodiploid fragmented DNA content, and the cleavage of the cell death associated caspase, caspase-3, in parallel with the cleavage of the widely accepted marker of cell death and a substrate for caspase-3, poly (ADP) ribose polymerase (PARP) [29]. The lethality of concentrations of mifepristone over 40 μM towards ovarian cancer cells was first suggested in 1996 by Rose and Barnea in OVCAR-3 and A2780 cells [44]. Yet, the results presented here are the first to demonstrate that the lethality of mifepristone monotherapy towards ovarian cancer cells is related to a caspase-associated apoptotic process. More importantly the toxicity of mifepristone did not discriminate among ovarian cancer cell lines with very different sensitivities to cisplatin, suggesting that mifepristone monotherapy could be useful for treating patients who have become platinum-resistant, for which the therapeutic alternatives have very disappointing outcomes [1,4,7].

The dose-dependent cytostatic and lethal effects of mifepristone towards ovarian cancer cells, which we will refer to globally as cytotoxicity, have been shown to also occur in breast cancer cells. A recent work using the MCF-7 breast cancer cell line illustrated that combination of mifepristone and the antiestrogen 4-hydroxytamoxifen had greater cytostatic and lethal activities than either monotherapy, whereas the lethality of the treatment was associated with genomic DNA fragmentation and cleavage of PARP [45]. In addition, it has been shown that MCF-7 made resistant to 4-hydroxytamoxifen also respond to mifepristone monotherapy undergoing apoptotic death [46]; finally, although at higher concentrations, mifepristone was also cytotoxic to progesterone receptor- and estrogen receptor-negative MDA-MB-231 breast cancer cells [47].

At present the role played by progesterone receptors in the cytotoxic activity of mifepristone remains unclear. We have reported that mifepristone has progesterone-like activity inhibiting growth of ovarian cancer cells [10]. Likewise in MCF-7 breast cancer cells it was shown that progesterone, instead of reversing the growth inhibitory activity of mifepristone, contributed to its growth inhibition effect [45]. A progesterone-like growth inhibitory action of mifepristone was also shown in estrogen-resistant, progesterone receptor expressing T47Dco breast cancer cells [48]. In addition, although mifepristone can bind to glucocorticoid and progesterone receptors with similar affinity [49], glucocorticoid receptors do not seem to mediate mifepristone action. In OV2008 ovarian cancer cells we could not mimic the growth inhibition effect of mifepristone when using equimolar concentrations of the glucocorticoid agonist dexamethasone (results not shown), whereas in MCF-7 breast cancer cells, dexamethasone was unable to reverse the inhibitory action of mifepristone [45]. Thus, it remains to be seen whether mifepristone utilizes cognate progesterone or glucocorticoid receptor-mediated endocrine mechanisms to drive its cytostatic and lethal effects on cancer cells.

While indirectly, in the present work we also analyzed whether the different p53 genetic status of the cells impacts the cytotoxicity of mifepristone. Normal function of the p53 tumor suppressor gene is associated with enhanced sensitivity to chemotherapy; several studies have suggested that loss of wild type p53 function may be a major cause of failure to respond to chemotherapy and radiotherapy [50,51]. Supporting this concept, in a study conducted utilizing the 60 cancer cell lines of the National Cancer Institute anticancer drug screen program, the majority of clinically active agents, including alkylating agents, antimetabolites, and topoisomerase inhibitors, tended to exhibit growth suppression more in the cell lines with normal p53 status than in the cell lines with mutant p53 status, with the exception of the anti-mitogenic agents [33]. This exception was confirmed in ovarian cancer cell lines where it was shown that the p53 status does not affect the sensitivity of the cells to the microtubule-stabilizing agent paclitaxel [52,53]. Therefore, although it is generally assumed that loss of normal p53 function can confer resistance to DNA-damage agents as a consequence of reduced susceptibility to apoptosis, the relevance of p53 mutations in chemosensitivity has exceptions and controversies particularly in terms of the potential functional activity of mutant p53 proteins [51,54-56]. Consequently, it is apparent that there are drugs for which the p53 background does not impact drug-sensitivity; in the present work we provide evidence that mifepristone behaves in that manner, inhibiting growth and triggering death of ovarian cancer cells which have been largely described as having different p53 genetic statuses (Table 1). For instance, Caov-3 cells that carry a point mutation leading to expression of an abnormal transcript encoding an inactive p53 [31,32], and SK-OV-3 cells that carry a single nucleotide deletion in the p53 gene and are not able to generate a p53 transcript [31,33,56,57], both have defects in their apoptotic machinery that associates with resistance to standard platinum therapy [58-60]. Conversely, OV2008 and A2780 cells expressing wild type p53 are very sensitive to cytotoxic drugs such as cisplatin [14,25], paclitaxel [61,62], or doxorubicin [63-65], rapidly undergoing apoptosis. Our data demonstrate that mifepristone, at concentrations beyond those used to achieve cytostasis has lethal activity triggering a caspase-associated apoptotic process in all six ovarian cancer cell lines studied regardless of their p53 genetic backgrounds and sensitivities to cisplatin. Although the potency of the growth inhibition by mifepristone was significantly higher in OV2008, OV2008/C13, and A2780 cells, all carrying wild type p53, when compared with SK-OV-3 and A2780/CP70 cells carrying p53 mutations, the calculated IC_50s _only ranged from 8 to 12 μM, suggesting that the inhibition is biologically relevant despite the p53 genetic backgrounds of the cells. Furthermore, the IC_50 _for mifepristone in Caov-3 carrying a mutant p53 gene is indistinguishable from that of the three wild type-carrying p53 cell lines (OV2008, OV2008/C13, and A2780), further supporting the notion that the p53 background is not relevant for the growth inhibition and the lethality triggered by mifepristone in ovarian cancer cells.

## Conclusion

The results obtained and summarized in Table 1 highlight the lack of correlation between the IC_50s _for mifepristone and the IC_50s _for cisplatin obtained for the ovarian cancer cell lines studied, and confirm the hypothesis that mifepristone growth inhibits ovarian cancer cells regardless of their sensitivities to cisplatin. Furthermore, we found that mifepristone, when used al lower concentration, shows cytostatic effects, whereas at higher concentration, it shows lethal effects towards all ovarian cancer cell lines studied, triggering a caspase-associated apoptotic death mechanism regardless of their degree of sensitivity to cisplatin and apparent p53 genetic status. The significance of this work lies in that it provides preclinical evidence suggesting that mifepristone monotherapy can be an alternative to treat ovarian cancers intrinsically resistant to clinically achievable doses of cisplatin, or recurrent ovarian cancer tumors which frequently have become platinum resistant and lack p53 function.

## Competing interests

The authors declare that they have no competing interests.

## Authors' contributions

EMF carried out most of the experiments and participated in the drafting of the manuscript. AAG participated in the design of the study and carried out and supervised some of the experiments. EES carried out some the experiments involving SK-OV-3 cells. CMT conceived the study and contributed to the writing of the manuscript. All authors read and approved the final manuscript.
